# «One Small Step for Mouse»: High CO_2_ Inhalation as a New Therapeutic Strategy for Parkinson’s Disease

**DOI:** 10.3390/biomedicines10112832

**Published:** 2022-11-06

**Authors:** Alexander D. Nadeev, Kristina A. Kritskaya, Evgeniya I. Fedotova, Alexey V. Berezhnov

**Affiliations:** Institute of Cell Biophysics of the Russian Academy of Sciences, Federal Research Center «Pushchino Scientific Center for Biological Research of the Russian Academy of Sciences», 142290 Pushchino, Russia

**Keywords:** neurodegeneration, Parkinson’s disease, neuroprotection, hypercapnia, experimental therapy

## Abstract

Parkinson’s disease (PD) is a ubiquitous neurodegenerative disorder for which no effective treatment strategies are available. Existing pharmacotherapy is aimed only at correcting symptoms and slowing the progression of the disease, mainly by replenishing dopamine deficiency. It is assumed that mitochondrial dysfunction plays a key role in the pathogenesis of PD. It has been suggested that activation of specific degradation of damaged mitochondria (mitophagy) may prevent cell death. An almost exclusive way to initiate mitophagy is acidification of intracellular pH. We attempted to implement transient brain acidification using two experimental therapy strategies: forced moderate physical activity and high CO_2_ inhalation. The beneficial effects of CO_2_ supplementation on behavioral aspects were demonstrated in a rotenone-induced PD model. Mice treated with CO_2_ restored their exploratory behavior and total locomotor activity lost after rotenone administration. Additionally, this treatment enabled the removal of impaired coordination. We have illustrated this therapeutic strategy using histological studies of brain sections to confirm the survival of nigrostriatal areas. These findings suggest that high CO_2_ inhalation presumably initiates mitophagy via transient brain acidification, and can treat PD-like symptoms in a rodent rotenone model of PD.

## 1. Introduction

Parkinson’s disease (PD) is socially significant, ubiquitous, and the second most common neurodegenerative disorder [1]. In the course of the pathological process, selective death of dopaminergic neurons in the midbrain is observed, mainly in the substantia nigra (SN) [2]. In turn, the loss of dopaminergic innervation of the striatum leads to impaired function of the basal ganglia, which are integral to the control of motor function, learning, and consciousness. Symptomatically, it is manifested in the development of motor disorders: tremor, hypokinesia, bradykinesia, muscle rigidity, and postural instability [2]. In addition to motor symptoms, various non-motor disorders may also develop, such as metabolic disorders and psychiatric disorders (e.g., psychosis, sleep disorders, depression). Still, the biochemical, genetic, and physiological prerequisites of Parkinson’s disease remain largely unclear. Existing treatments are symptomatic and have low efficacy [3]. Drugs are used to compensate for dopamine deficiency in the basal ganglia by increasing the content of this neurotransmitter, activating dopamine receptors, and inhibiting dopamine decay [3]. Thus, the search for new therapeutic approaches in the treatment of PD, based on an understanding of the cellular mechanisms of neurodegeneration development, is urgent.

Numerous studies have investigated the molecular basis of neurodegeneration in PD. Among them are α-syn pathology [4] and mitochondrial disorders [5], apoptotic and non-apoptotic programmed cell death [6], autophagic regulation [7], endoplasmic reticulum dysfunction [8], and calcium homeostasis impairment [9]. However, the issue of establishing the chronological order and mutual influence of these events in the development of neurodegeneration still remains unresolved.

One of the insights into understanding the neurogenerative mechanisms stems from the action of some toxic compounds, which may exhibit motor and nonmotor symptoms resembling PD. Exposure to neurotoxins such as 6-OHDA, paraquat, MPTP, and rotenone causes death of dopaminergic neurons in the SN. Their mechanism of action is to disrupt mitochondrial function and increase oxidative stress [10,11,12]. Rotenone is a pesticide that is widely used to kill pests such as insects and fish. This substance binds to and inhibits mitochondrial ETC complex I. Rotenone is lipophilic and easily penetrates all cells [12]. Rotenone exposure causes selective degeneration of nigrostriatal dopaminergic neurons, accompanied by the formation of Levy bodies [13]. When administered to rodents, rotenone causes the loss of neurons in the SNpc, depletion of dopamine in the striatum, and manifestation of motor and non-motor symptoms [11]. The rotenone model has been applied not only to animals, but also to cell cultures [14].

Another clue was obtained when studying the mutations that cause inherited forms of PD. Familial forms of PD are caused by mutations in a number of genes encoding proteins such as α-synuclein, PINK1, Parkin, DJ-1, LRRK2, VPS35, and EIF4 G1 [9,15,16,17,18,19]. These proteins are associated with vesicular transport [4,15,16], calcium homeostasis [9], cellular antioxidant systems [15], mitochondrial functioning [17], and auto/mitophagy regulation [18,19].

Thus, exploring toxic models and dissecting familial forms of PD presents the evidence of impaired functioning of mitochondria in midbrain neurons, including impaired mitophagy, i.e., degradation of damaged mitochondria with the participation of lysosomes [5,20]. The accumulation of dysfunctional mitochondria can lead to cell death. It is assumed that the activation of mitophagy can prevent cell death and return to normal functioning [21]. However, despite intensive studies of the molecular mechanisms of mitophagy, little is known about the methods of induction of this process. It has been shown that acidification of the cytosol, including short-term acidification, causes the activation of autophagy and mitophagy in cells [22]. Previously, we have demonstrated that lactate and pyruvate are able to recover mitochondrial function, lost after MPTP administration, by inducing mitophagy via reducing intracellular pH [23]. Lactate concentrations in the organism can be dramatically increased during physical activity [24]. It has also been shown that the brain absorbs lactate in proportion to the concentration in arterial blood [25]. On this basis, we hypothesized that exercise may indirectly induce mitophagy in midbrain neurons.

Alternatively, an increase in the concentration of carbon dioxide (up to 5–20%), in the inhaled air was shown to induce a reversible acidification of brain cells [26,27]. Consequently, this can promote mitophagy. Thus, we created the idea that forced inhalation of a gas–air mixture with a high content of CO_2_ can activate mitophagy and promote neuronal survival.

In this work, we aimed to test two experimental therapy approaches. The first one was moderate physical activity treatment, based on the neuroprotective potential of lactate. The second was high CO_2_ inhalation as a novel strategy to treat PD in a rotenone rodent model.

## 2. Materials and Methods

### 2.1. Animals

Healthy male CD-1 mice 12 months in age, and weighing 32~64 g, were housed in polypropylene cages under hygienic conditions and were provided free access to standard animal feed and water throughout the treatment period.

All animal studies were performed in accordance with the legal requirements listed in ICB RAS (Institute of Cell Biophysics Russian Academy of Sciences) Manual for Working with Laboratory Animals (approved by the Commission on Biosafety and Bioethics of ICB RAS, Protocol No. 57, 30 December 2011). All experimental protocols in this study were approved by the Commission on Biosafety and Bioethics of ICB RAS (Permission No. 2, 12 June 2020). Experimental protocols were carried out according to Act708n (23 August 2010) of the Russian Federation National Ministry of Public Health, which states the rules of laboratory practice for the care and use of laboratory animals, and the Council Directive 2010/63 EU of the European Parliament (22 September 2010) on the protection of animals used for scientific purposes.

### 2.2. Experimental Design and Induction of Parkinsonism

A cohort of 35 mice was initially divided into 2 (I and II) groups: control (*n* = 9) and rotenone-treated (*n* = 26). Rotenone (Sigma, St. Louis, MO, USA), dissolved in olive oil, was injected intraperitoneally 5 times a week for 6 weeks at a dose of 2 mg/kg body weight; control animals received olive oil vehicle. After 14 days of rotenone injections, two (running and CO_2_ inhalation, see below) treatment procedures started, and the animals were finally divided into 6 experimental groups.

1. Control mice (I) (received only vehicle) were divided randomly in 3 groups:
  I-0 negative control (*n* = 3)
  I-a moderate physical activity treatment (*n* = 3)
  I-b CO_2_ treatment (*n* = 3).
2. Rotenone treated (II) mice were randomly divided into 3 groups:
  II-0 negative control (*n* = 9)
  II-a moderate physical activity treatment (*n* = 9)
  II-b CO_2_ treatment (*n* = 8).

### 2.3. Experimental Therapy Treatments

#### 2.3.1. Forced Moderate Physical Activity

Three times a week, animals were exposed to forced running on a treadmill. Each running practice consisted of 3 runs: for 1 min at a 4 m/min rate, followed by 1 min break, then 2 2 min runs at a 10 m/min rate separated by a 1 min break.

#### 2.3.2. CO_2_ Inhalation

Three times a week, animals were placed in a closed glass cylinder, filled with 20% CO_2_ atmosphere for 2 min.

### 2.4. Behavioral Evaluation

Mice were subjected to pretraining on all the behavioral parameters before the start of any treatment. The experimental laboratory units were cleaned after each test with an aqueous solution containing ethanol (40% *v*/*v*) to avoid possible biasing effects from the previous animal. The tests were performed before the rotenone injections, after 14 days of rotenone exposure, and at the end of each from 4 weeks of experimental therapy.

#### 2.4.1. Beam Walking Test

The beam test was performed as described by Fleming [28]. The beam used had the following characteristics: length 1 m, width of lower beam 5.5–2.5 cm, width of upper beam 4–0.5 cm, beam height 1 cm, above table height 40 cm. Beam was equipped with a mirror. All animal performances were filmed, and number of paw slips, i.e., walking mistakes, were calculated visually on a video. All animal attempts were done in triplicate to record the sum score of walking mistakes.

#### 2.4.2. Cylinder Test

The exploratory behavior and total locomotor activity were assessed by the cylinder test as described by Fleming [28]. Each CD-1 mouse was placed into a glass cylinder for 3 min, and the number of rears per minute was recorded. This included the full rear when the animal was vertical with the forelimbs off the bottom or with one or both the forelimbs touching the cylinder.

### 2.5. Histology Preparations

Mice were anaesthetized with 40 mg/kg zolazepam (Zoletil^®^, Virbac, Carros, France), then animals were decapitated, the brains were extracted and fixed in Carnoy’s solution (ethanol–chloroform–acetic acid 6:3:1, respectively), encased in paraffin. Slices of 10 μm thickness were prepared from the blocks. The state of neurons in the substantia nigra (SN) was determined by staining brain slices with cresyl violet (Fluka Chemical, Sigma-Aldrich, St. Louis, MO, USA) (Nissl staining). Histological preparations were analyzed using a Nikon Eclipse TS100 optical microscope (Nikon, Tokyo, Japan).

### 2.6. Primary Rat Cortical Neuroglial Culture

Primary neuroglial brain cortex culture was obtained from newborn male (P1–3) Sprague Dawley rats (also approved by institutional statement). The pups were decapitated; the brains were extracted and placed in a 60 mm Petri dish in cold sterile HBSS (Paneco, Moscow, Russia) solution on ice. Then, the meninges were removed; the tissue was placed in a microcentrifuge tube in a cold Versene solution (Paneco) and was dissected in small pieces (1 mm). Then, the medium was replaced with a 0.1% trypsin solution incubated for 15 min at 37 °C. After that, trypsin was washed 3 times with DMEM medium (Sigma-Aldrich) with 10% FBS (Sigma-Aldrich), gently pipetting the tissue with a 1000 μL tip until a homogeneous suspension was formed. Next, the cells were placed on round coverslips (25 mm), coated with polyethyleneimine, placed in 35 mm Petri dishes, and left for 30 min for cell attachment. After that, culture medium was replaced with 1.5 mL Neurobasal A medium (Gibco, Grand Island, NY, USA) containing 2% supplement B27 (Gibco), 1 mM L-glutamine (Gibco), and 7.5 μg/mL gentamicin (Gibco), and cells were incubated for 9–14 days at 37°C and 5% CO_2_.

#### Cell Viability

Microscope imaging experiments were performed at a temperature of 28 °C. The working medium (HBSS) contained (in mM) the following: 138 NaCl, 1.3 CaCl_2_, 0.4 MgSO_4_, 0.5 MgCl_2_, 5.3 KCl, 0.45 KH_2_PO_4_, 4 NaHCO_3_, 0.3 Na2HPO_4_, 10 glucose, and 20 HEPES (pH = 7.3). To assess cell viability, the Hoechst 33342 (Thermo Fisher Scientific, Waltham, MA, USA) (2 μg/mL, 10 min) and propidium iodide (Thermo Fisher Scientific) (2 μg/mL, 10 min) were used. The freely penetrating Hoechst 33342 label DNA in all cells and propidium iodide are able to penetrate only into damaged cells, indicating necrosis. The fluorescence of Hoechst 33342 and propidium iodide was evaluated using a Leica DMI6000 B inverted microscope (Leica Microsystems, Wetzlar, Germany) using a 20× objective using standard filter sets for DAPI and Texas Red, respectively.

In the experimental procedure, cells were incubated with 1 mg/mL rotenone for 12 h, then 10 mM sodium lactate was added to the medium for another 24 h, after which the cells were washed and the percentage of necrotic cells in the field of view was calculated.

Cell culture experiments were done in three biological triplicates, with a minimum of three fields of view calculated for each coverslip.

### 2.7. Statistical Analysis

Statistical analysis was processed using GraphPad Prism 6.0 software (San Diego, CA, USA). Statistical data were expressed as mean ± SD. After the Shapiro–Wilk normality test, differences between groups were analyzed using Student’s t-test, Mann–Whitney test, or one-way ANOVA with Bonferroni correction. Kaplan–Meier survival analysis was also performed using GraphPad Prism 6.0.

## 3. Results

### 3.1. Action of Sodium Lacatate on Rotenone Exposed Neurons

**Figure 1:**
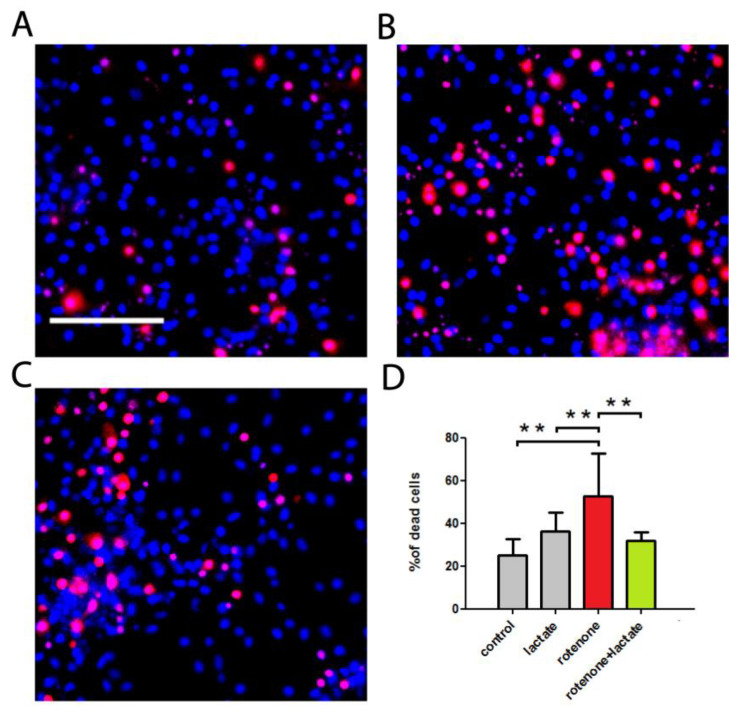
The effect of sodium lactate on rotenone-induced cell toxicity. Rotenone was added to the rat brain cell culture 36 h before measurements; 12 h after the addition of rotenone, sodium lactate was added. (**A**–**C**) Representative images of cells in primary cortical neuroglial culture stained with Hoechst 33342 (blue) and propidium iodide (red). (**A**) Control conditions (**B**) After 36 h of 1 mg/mL rotenone exposure (**C**) Rotenone in combination with 10 mM sodium lactate. Scale bar: 100 μm. (**D**) Percentage of nonviable cells, *n* = 3 experiments, ** *p* < 0.01.

At first, we aimed to evaluate the protective effect of sodium lactate-induced acidification in the PD cell model. Rat neuroglial brain culture cells were used for this purpose. Cells were incubated with rotenone for 12 h, then sodium lactate was added for another 24 h, after which the cells were washed and the percentage of necrotic cells was estimated. Cell culture microphotographs are presented in Figure 1A–C, and the results are summarized in Figure 1D. It was found that rotenone caused more than half of the cells to die (52.80 ± 19.71%) in the rat neuroglial brain culture within 36 h (Figure 1B). Application of 10 mM sodium lactate significantly reduced rotenone-induced cell death. Thus, under the effect of 10 mM sodium lactate against rotenone in the neuroglial culture, the percentage of nonviable cells was 31.99 ± 4.02%. No significant independent effect of sodium lactate on cell viability was detected: 36.27 ± 8.95 versus 24.99 ± 7.65 % of nonviable cells in the control group.

Here we provide experimental evidence that sodium lactate may serve as neuroprotector against rotenone neurotoxicity. Its beneficial action against MPTP toxicity, and in the familial forms of PD through the activation of mitophagy, was shown in our previous studies [22,23]. Further, in the course of this study we aimed to test this concept at the organism level.

### 3.2. Development of PD-like Symptoms in Rotenone Exposed Mice

**Figure 2:**
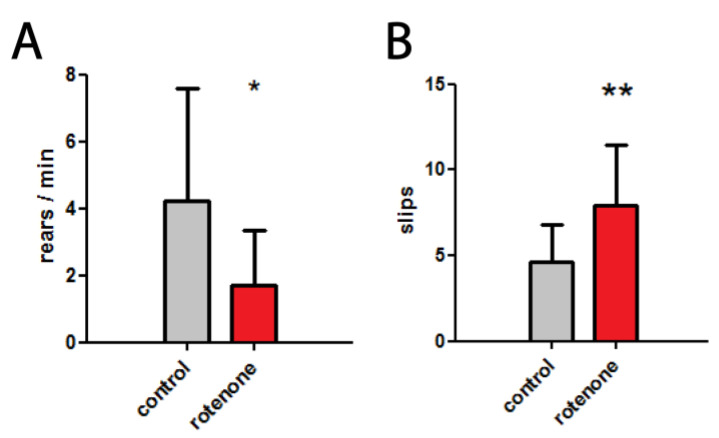
Development of PD-like symptoms in mice after 14 days of rotenone exposure. (**A**) The number of rears per minute in a cylinder test, * *p* < 0.05 (**B**) The number of paw slips (mistakes) in a beam walking test, ** *p* < 0.01.

As described in Materials and Methods, at first, mice were divided into two groups: control and rotenone exposed. After 14 days of rotenone exposure, we observed a significant decrease in motor balance and coordination as well as a significant decrease in exploratory behavior (activity). The number of rears per minute in a cylinder test was 4.22 ± 3.37 in the control group, compared with 1.70 ± 1.66 in the rotenone treated group (Figure 2A). The number of paw slips during the beam walking test was 7.96 ± 3.52 in the rotenone group, and 4.62 ± 2.20 in the control group (Figure 2B). These changes recorded after two weeks of rotenone administration became the basis for subsequent therapeutic procedures: moderate physical activity and CO_2_ inhalation.

### 3.3. Moderate Physical Activity Treatment

**Figure 3:**
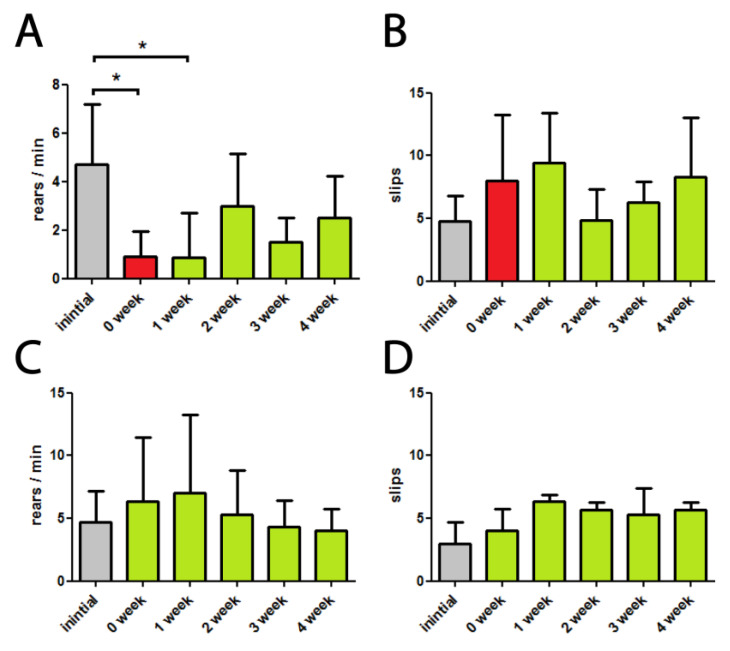
Effects of moderate physical activity treatment on rotenone-injected (**A**,**B**) and control (**C**,**D**) mice. 0-week denotes the beginning of treatment, after 2 weeks of rotenone exposure. (**A**–**C**) The number of rears per minute in a cylinder test, * *p* < 0.05 (**B**–**D**) The number of paw slips (mistakes) in a beam walking test.

Untreated and rotenone exposed mice were subjected to moderate running activity three times per week. At the end of each of the four weeks of treatment, physiological tests were performed. The results for groups II-a (rotenone treated) and I-a (control) are summarized in Figure 3.

Exploratory behavior assessed by cylinder test remained strongly repressed through the experimental period (Figure 3A). Beam travel mistakes did not differ significantly from before treatment level indicating no positive changes in the motor balance and coordination state (Figure 3B). Nevertheless, we observed positive tendencies of recovering of locomotion and coordination after 2–4 weeks of this treatment, but they should probably be explained by intermediate mortal cases. After four experimental weeks, the total mortality in this group was 55% (50 % in control II-0 group), and the average weight loss was 9.2% (4.9 % in control II-0 group).

### 3.4. High CO_2_ Treatment

**Figure 4:**
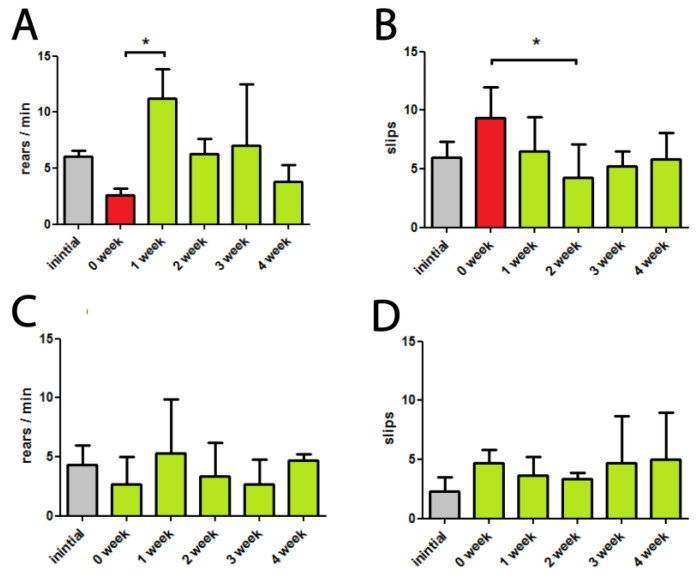
Effects of high CO_2_ inhalation treatment on rotenone-injected (**A**,**B**) and control (**C**,**D**) mice. 0-week denotes the beginning of treatment, after 2 weeks of rotenone exposure. (**A**–**C**) The number of rears per minute in a cylinder test, * *p* < 0.05 (**B**–**D**) The number of paw slips (mistakes) in a beam walking test, * *p* < 0.05.

Animals from both the control and rotenone-injected groups were exposed to 20% CO_2_ inhalation three times per week. At the end of the weeks 1 to 4 animals performed physiological tests. The results are presented in Figure 4.

Firstly, we can notice that the CO_2_ treatment resulted in increased exploratory behavior in the first week, which was lost after rotenone administration; however, it enabled a recovery of it in later weeks (Figure 4A). In terms of values, the number of rears per minute in the first week was two times higher compared to the initial level, was close to it in the second and third week, and decreased partially in the fourth week.

Secondly, the CO_2_ treated group showed a significant decrease in the beam travel mistakes compared with before treatment level (Figure 4B). Most vividly, there was a two-time significant decrease in the 2nd week of treatment. During the other weeks, the number of mistakes in the CO_2_ group also was fewer than the before therapy values.

Here we can remark that in the respective CO_2_ treated control group (I-b) there were not any trends or significant changes in the above-described physiological tests (Figure 4C–D).

After four experimental weeks, total mortality in this group was 33%, and the average weight loss was 11.7%.

**Figure 5:**
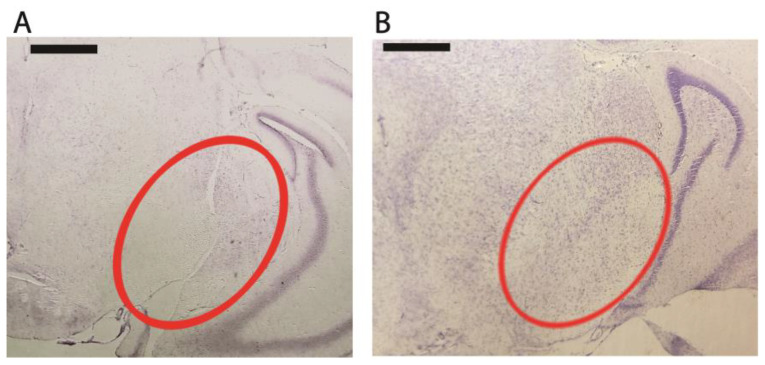
Effects of high CO_2_ inhalation. Nissl staining of paraffinized coronal brain slices of mice exposed to rotenone. (**A**) untreated (**B**) CO_2_-treated. Scale bar: 500 μm. SN is circled in red.

For additional verification, we also performed Nissl staining of paraffinized slices of mouse brain. Pictures from rotenone administrated and rotenone administrated, CO_2_-treated mice are presented in Figure 5. We observed a substantial neuron loss in mice treated with rotenone (Figure 5A). At the same time, we can see that the CO_2_ treated animal, unlike the rotenone-exposed one, preserved its neurons in the substantia nigra (Figure 5B).

### 3.5. Survival Analysis

**Figure 6:**
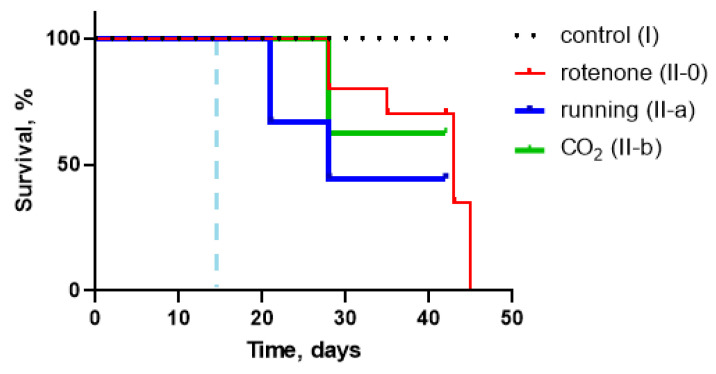
Survival Kaplan–Meier curves for control (I), rotenone exposed (II-0), moderate physical activity (running) treated (II-a), and CO_2_-treated (II-b). No significant differences were found between groups (*p* > 0.05). The dashed blue line indicates therapy start point.

As previously mentioned, survival rates (% of survived animals at the end of the experiment) were as follows: 50% in the rotenone-injected (II-0) group, 54% in the exposed to moderate physical activity group (II-a), 33% in the CO_2_-treated group (II-b). To confirm if there was any significant effect of our therapy procedures on experimental animals’ survival we used a Kaplan–Meier plot with subsequent analysis. Results are presented on Figure 6. Calculations showed no difference between survival curves.

## 4. Discussion

Parkinson’s disease is the second most common neurodegenerative disorder worldwide [1]. Currently, the etiopathology of PD is poorly understood. However, the selective oxidative stress in the SN is the earliest and the most widely accepted hypothesis for the etiopathology of PD [29]. In particular, oxidative stress in PD could occur due to decreased activity of mitochondrial complex I [30]. Conveniently, rotenone as a complex I inhibitor, can cause nigrostriatal degeneration in rodents [31]. The extent of nigrostriatal dopaminergic loss can be assessed by very sensitive motor behavioral tests.

In this study, rotenone-injected mice showed decreased locomotor activity in a cylinder test and impaired coordination during the beam travel test. These changes, recorded after two weeks of toxicant administration, became the basis for subsequent therapeutic procedures.

The beneficial effects of physical activity have been known since ancient times. The unknown and diverse factors underlying it are being intensively investigated. For instance, a recent study provides a link between the cognitive benefits of exercise and the increase in brain-derived neurotrophic factor (BDNF) [32]. Another described mechanism includes oligodendrogenesis mediated by nerve growth factor (VGF) [33]. Previously, with our participation, it was shown that acidification of the cytosol by lactate and pyruvate can activate mitophagy and protect cells in the familial form of PD [34]. We hypothesized that increased after physical exercise, blood lactate could provide transient acidosis in the midbrain region, reducing oxidative stress via initiation of mitophagy.

In our experiments, moderate physical activity treatment did not result in significant recovery changes in the tested neurobehavioral parameters. Although we observed positive tendencies of recovering locomotion and coordination in 2–4 weeks of this treatment, they should probably be explained by intermediate mortal cases.

Here we can note that the total mortality after six weeks, 50% in the control group (II-0), was unexpectedly high regarding the common protocol of rotenone PD model [31,35,36]. This might be associated with the use of relatively old experimental animals. To the best of our knowledge, no PD toxic model studies have been performed on rodents of such age. This particular feature may also explain relatively fast development of PD-like symptoms. At the same time, regarding the age-specific profile of PD [37] the old age of mice could be advantageous for therapeutic strategy development.

While on the subject of therapeutic strategies, our second therapeutic procedure, consisting of forced high CO_2_ inhalation, demonstrated promising and satisfactory results. Based on the experimental evidence that high CO_2_ inhalation provides transient acidosis in brain [26,27], we hypothesized that this treatment would promote mitophagy to remove damaged mitochondria and to enable cell survival. Mice exposed to a 20% CO_2_ atmosphere three times a week showed no signs of impaired coordination assessed in the beam walking test throughout 4 weeks of experimental therapy with a significant difference from before therapy level on the second week. Additionally, in the cylinder test, we observed restored, and even elevated in the first week, total locomotor activity decreased after rotenone administration. Applied together, these tests demonstrate promising beneficial effects of CO_2_ supplementation on behavioral indicators, which were also validated by histological studies of brain sections to confirm the survival of certain areas.

Therefore, we provide primary experimental verification of our proposed, previously unused therapeutic approach. Firstly, we have demonstrated that high CO_2_ inhalation can treat PD-like symptoms in a rodent rotenone model of PD, presumably via transient brain acidification and thus initiation of mitophagy. The undoubted advantages of this therapeutic approach are the simplicity of its implementation, non-invasiveness of the impact, and low cost of therapeutic materials.

## 5. Conclusions

Our findings demonstrate that CO_2_ inhalation attenuated rotenone-induced locomotor and coordination impairment and SN damage in mice. The recovering effect of CO_2_ inhalation might occur through transient brain acidification that initiates mitophagy and enables neuron survival. Considering further research, this may be of high clinical significance.

## Data Availability

The data presented in this study are available on request from the corresponding author.
